# Commentary: Thyrotropin Stimulates Differentiation Not Proliferation of Normal Human Thyrocytes in Culture

**DOI:** 10.3389/fendo.2017.00214

**Published:** 2017-08-25

**Authors:** Aglaia Kyrilli, Sabine Paternot, Françoise Miot, Bernard Corvilain, Gilbert Vassart, Pierre P. Roger, Jacques E. Dumont

**Affiliations:** ^1^IRIBHM, Université libre de Bruxelles, Brussels, Belgium; ^2^Department of Endocrinology, Erasme University Hospital, Université libre de Bruxelles, Brussels, Belgium

**Keywords:** TSH, cyclic AMP, human thyrocytes, *in vitro* proliferation, *in vivo* proliferation

We want to express our concern about the article “Thyrotropin stimulates differentiation not proliferation of normal human thyrocytes in culture” (1) published recently in this journal. We show that this study draws the wrong conclusions from negative experiments performed in conditions that were previously shown to be inadequate, ignoring fundamental experimental articles on the subject by our group and others (2–11) [see also reviews (12–16)]. The inference that TSH would have no proliferative effect *in vivo* on human thyrocytes could have negative clinical consequences, as it would negate the fundamental rationale of thyroxine treatment post thyroidectomy for differentiated thyroid cancer or for goiter.

Hereby, we present a compilation of published data showing that TSH, through cAMP, stimulates the proliferation of human thyrocytes in primary culture; we analyze the reasons for Morgan’s failure to reach that conclusion; and we summarize the extensive literature on the subject including *in vivo* and *in vitro* studies.

We present in Figure 1 the compilation of proliferation results obtained in 35 independent primary cultures used in studies related to previous publications from 1987 (6–9, 17, 18). Our protocol complied with ULB ethical rules and was approved by local ethics committee. Cell culture conditions and proliferation measurement by BrdU or [^3^H] thymidine incorporation are precisely described (7, 8). Not one experiment with available results obtained with these proliferation assays was excluded. Although with considerable biological variability, inherent to *ex vivo* culture of human tissue from surgical origin, we observed that addition of TSH (0.3 mU/ml), forskolin (10 µM), or EGF (25 ng/ml) plus fetal bovine serum (FBS) 10% to the culture medium significantly increased thyrocyte proliferation rate from median 9.2% (percentiles 5–95: 1.7–25.2) in control conditions to 25.3% (3.24–55.2), 18.0% (3.8–44.15), and 21.3% (9.1–49.8) in cells stimulated by TSH, forskolin, and EGF + FBS, respectively. TSH stimulation was also perfectly reproduced using highly purified TSH (8). Overall, it was as strong as the maximal cAMP-independent mitogenic stimulation by EGF + FBS.

**Figure 1:**
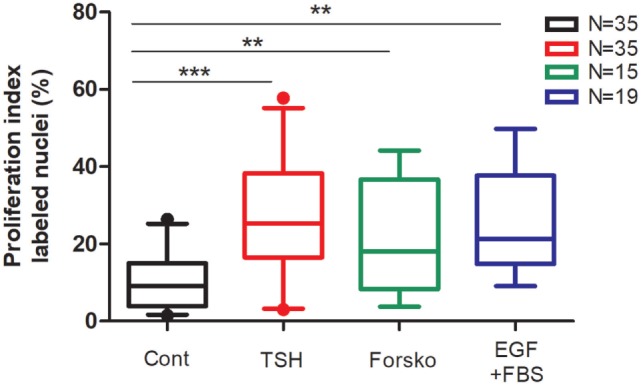
Compilation of human thyrocyte proliferation stimulated by TSH, forskolin, EGF, and fetal bovine serum (FBS) experiments. Human thyroid follicles were prepared by collagenase digestion and seeded in a medium containing Dulbecco’s Modified Eagle’s Medium, Ham’s F-12 nutrient mixture, and MCDB 104 medium (2:1:1, by vol), 1 mM sodium pyruvate, 2 mM glutamine, 5.625 µg/ml insulin, 2.5 mg/ml human transferrin, 40 µg/ml ascorbic acid, 2.5 µg/ml amphotericin B, 100 U/ml penicillin, and 100 µg/ml streptomycin (8). To ensure optimal spreading of the follicles 1% FBS was added for the first 24 h of culture. The ability of human thyrocytes to proliferate in primary cultures was measured by DNA incorporated BrdU or [^3^H] thymidine (incorporation time 24 h between days 4 and 5). TSH (0.3 mU/ml), forskolin (Forsko; 10 µM), and EGF (25 ng/ml) + FBS 10% added at day 1 significantly increased the proportion of cell undergoing cell cycle progression. Data (box and whiskers) represent median, quartiles, and 5–95 percentiles. *N* = number of experiments. Statistical significance was evaluated using an unpaired *t*-test with Welch’s correction: **p < 0.001, ***p < 0.0001.

There is no doubt that TSH activates thyrocyte proliferation through cAMP in humans *in vivo* as it does it in experimental animals. It accounts:

(1) for autonomous adenomas and familial non-immune hyperthyroidism caused by TSHR mutations activating the TSH receptor effect on cAMP (3, 19–22);
(2) for the effect on thyroid growth of TSAb in Graves’ disease which, through the TSH receptor, activates the cAMP cascade (5, 23, 24);
(3) for thyroid reduction following suppressive thyroxine therapy (25);
(4) for hyperthyroidism resulting from pituitary hypersecretion of TSH, in the case of TSH secreting adenomas (26). However, in this case TSH activates both the cAMP and IP3-Ca2+ cascades (27).

These conclusions have been validated in transgenic mice *in vivo*, in which the forced thyrocyte expression of the adenosine A2 receptor (28), activated Gsα (29), or cholera toxin (30), all constitutively activating adenylate cyclase, also leads to hyperthyroidism and goiter. Finally, the mitogenic action of TSH was also demonstrated in xenotransplants of human thyroid tissue in mice (31, 32).

With an optimal culture protocol [1% FBS for follicles seeding, defined medium, presence of IGF1, or insulin (4, 11, 18, 33)] and thyroid material [not goiter, not from old people, not previously frozen, subcultivated, and/or cells exposed to high serum concentrations (34–38)], two doublings of the cell population in 9 days were demonstrated in TSH-stimulated cells *in vitro* (8).

The possible causes of false negative in *in vitro* experiments as obtained by Morgan et al. are contradicted by positive results obtained for years in several laboratories and could be explained as follows:

(1) Morgan et al. (1) only used thyrocytes that were initially propagated in the presence of high serum concentration. Both in human thyrocytes (4) and in canine thyroid primary cultures (39), this suffices to irreversibly abolish the mitogenic response to TSH and cAMP as assayed by cell counting and/or DNA synthesis measurements, without impeding the mitogenic response to growth factors or differentiation effects of TSH;
(2) seeding of isolated cells instead of follicles;
(3) inadequate basal culture medium. Dulbecco’s Modified Eagle’s Medium (DMEM) alone does not support thyrocyte proliferation after serum deprivation;
(4) only use of cell counting which is a good but poorly sensitive measurement in which proliferation can be compensated by cells loss, especially in inadequate culture condition like DMEM medium.
(5) study of “normal” tissue from thyroidectomy performed for thyroid cancer, Graves’ disease or multinodular goiter [our 1988 study (8) mostly used thyroid tissue from traffic accident victims]. The proliferative effect of cAMP is a characteristic of normal differentiated thyroid cells which is much decreased in dedifferentiated cells and even more so in cancer cells (40).

Surprisingly, Morgan et al. (1) did not even attempt to reproduce experimental conditions that were successful in other laboratories.

Besides the *in vitro* arguments, the rationale for treating patients with papillary or follicular carcinoma with suppressive doses of thyroxine is also based on multiple clinical studies with arguments summarized in a review by Biondi et al. (41):

(1) hypothyroidism stimulates neoplastic thyroid cell growth;
(2) patients treated with L-T4 have less recurrence and cancer-related death than untreated patients;
(3) TSH suppression is an independent factor of slower progression at least in patients with high-risk papillary thyroid cancer.

Even if all these studies are retrospective or prospective but not randomized, they strongly suggest that TSH may stimulate cancer thyroid cell growth. In the last American thyroid Association guidelines (Recommendation 70), TSH suppression below 0.1 mU/l is recommended in patients with structural incomplete response (strong recommendation, moderate-quality evidence), which is among the strongest recommendations that are made in a field where prospective studies are rare. Questioning this recommendation on the base of *in vitro* data not obtained by other groups is presumptuous and asking for a double-blind prospective study is unrealistic given the number of patients that should be included and the duration of follow-up to be expected for a slowly evolving disease.

The TSH effect on growth of human thyroid cells should remain the scientific basis of TSH suppression treatment with thyroxine after thyroidectomy for differentiated thyroid cancer and a possibility for the temporary treatment of goiter.

## Author Contributions

AK and SP have performed experiments and written the manuscript. FM, BC, GV, PR, and JD have performed a thorough literature research and reviewed and commented the manuscript.

## Conflict of Interest Statement

The authors declare that the research was conducted in the absence of any commercial or financial relationships that could be construed as a potential conflict of interest.
